# An Intronic Polymorphism in *couch potato* Is Not Distributed Clinally in European *Drosophila melanogaster* Populations nor Does It Affect Diapause Inducibility

**DOI:** 10.1371/journal.pone.0162370

**Published:** 2016-09-06

**Authors:** Valeria Zonato, Giorgio Fedele, Charalambos P. Kyriacou

**Affiliations:** Department of Genetics, University of Leicester, Leicester, LE1 7RH, United Kingdom; Biomedical Sciences Research Center Alexander Fleming, GREECE

## Abstract

*couch potato* (*cpo*) encodes an RNA binding protein that has been reported to be expressed in the peripheral and central nervous system of embryos, larvae and adults, including the major endocrine organ, the ring gland. A polymorphism in the *D*. *melanogaster cpo* gene coding region displays a latitudinal cline in frequency in North American populations, but as *cpo* lies within the inversion *In(3R)Payne*, which is at high frequencies and itself shows a strong cline on this continent, interpretation of the *cpo* cline is not straightforward. A second downstream SNP in strong linkage disequilibrium with the first has been claimed to be primarily responsible for the latitudinal cline in diapause incidence in USA populations.Here, we investigate the frequencies of these two *cpo* SNPs in populations of *Drosophila* throughout continental Europe. The advantage of studying *cpo* variation in Europe is the very low frequency of *In(3R)Payne*, which we reveal here, does not appear to be clinally distributed. We observe a very different geographical scenario for *cpo* variation from the one in North America, suggesting that the downstream SNP does not play a role in diapause. In an attempt to verify whether the SNPs influence diapause we subsequently generated lines with different combinations of the two *cpo* SNPs on known *timeless* (*tim)* genetic backgrounds, because polymorphism in the clock gene *tim* plays a significant role in diapause inducibility. Our results reveal that the downstream *cpo* SNP does not seem to play any role in diapause induction in European populations in contrast to the upstream coding *cpo* SNP. Consequently, all future diapause studies on strains of *D*. *melanogaster* should initially determine their *tim* and *cpo* status.

## Introduction

*couch potato* (*cpo*) encodes a RNA recognition motif and a nuclear localization sequence and was first identified in a screen for genes involved in *D*. *melanogaster* central nervous system development [1,2]. The name was chosen because of the overall hypoactive behavior and sluggishness conferred by mutations in this gene. Viable mutant flies are characterised by delayed development, compromised flight response, abnormal phototaxis, geotaxis and ether recovery, and increased seizure susceptibility [2,3]. CPO is expressed in the peripheral and central nervous system of embryos, larvae and adults, as well as in other tissues such as the midgut, glia, salivary glands and is also reported to be expressed in the ring gland, the major endocrine organ in *D*. *melanogaster* [1,4].

*cpo* was also identified in a QTL screen as a putative candidate gene involved in *D*. *melanogaster* diapause [5], which is a seasonal response that leads to *D*. *melanogaster* adult females arresting ovarian development at temperatures below 13°C. This phenotype can be enhanced, at least in some strains, by shorter photoperiods [6,7]. Schmidt et al (2008) observed that higher diapause incidence correlated with elevated levels of *cpo* expression, an observation that resonates with similar findings for diapause initiation in *D*. *montana* [8]. In *D*. *melanogaster cpo*, two non-synonymous SNPs at residues 356 (Alanine to Valine, *C/T cpo*^*Ala356Val*^) and 462 (Isoleucine to Lysine, *T/A*, *cpo*^*Ile462Lys*^) were initially identified, with the latter displaying the strongest association with diapause [5]. *cpo*^*356Val*^ increases in frequency with latitude in North America and given the strong linkage disequilibrium between the two *cpo* SNPs, a rather steeper *cpo*^*462Lys*^ cline was also detected by sequencing the *cpo* region only in two populations at the extremes of the latitudinal range [5], but it was argued that *cpo*^*462Lys*^ represented the focal *cpo* polymorphism for diapause variation on this continent. Complementation analysis indeed suggested the involvement of *cpo* in determining diapause influence [5] and focused on residue 462. However, given the strong LD with site 356, it was not clear whether site 356 might have been contributing to the phenotype observed [5]. In addition, at the time the study was performed, it was not known that the second chromosome *ls/s-tim* polymorphism also played a significant role in diapause inducibility [9,10]. The clines at these two *cpo* sites remained largely stable in fly populations collected 12–13 years later [11]. Subsequent studies, aimed at identifying spatially varying loci, identified *cpo* as an Fst outlier in both American [12] and Australian populations [13]. However the finding ceased to be significant when correction for multiple testing was applied [14].

Not surprisingly, diapause levels varied in flies collected along the eastern coast of the USA, with the incidence of this trait predictably increasing with latitude [15] supporting similar earlier findings [16]. Diapause incidence has also been shown to vary throughout the year in American populations of *D*. *melanogaster* [17], and *cpo*^*Ile462Lys*^ was found to display the strongest association with month of collection [11].

In eastern Australia *cpo*^*462Lys*^ also increases significantly with latitude, but this appears to be driven by its strong linkage disequilibrium with the inversion, *In(3R)Payne*, within which *cpo* is located [18]. Indeed, *In(3R)Payne* also shows strong clinal distribution in both North America and Australia [19,20] complicating any interpretation of *cpo* clines. Furthermore, unlike American populations, *cpo*^*Ile 462Lys*^ does not appear to account for differences in diapause levels in different Australian populations [18]. We therefore sought to re-examine the putative *cpo* clines in *D*. *melanogaster* populations from Europe where colonization by this species from its roots in sub-Saharan Africa is believed to have occurred ~14 Kya since the last glaciation [21].

An intriguing scenario is also raised in Europe due to an important polymorphism in the clock gene *timeless* (*tim*). A naturally occurring mutation in the TIM N-terminal, recruited an addition 23 residue exon from an upstream methionine codon giving rise to a novel L-TIM isoform in addition to the ancestral S-TIM isoform initiated from the downstream methionine [22]. This *ls-tim* allele is believed to have arisen in southern Italy a few thousand years ago at most and has spread by directional selection because it is better adapted to European seasonal changes than the ancestral *s-tim* allele [9,10]. One of these dramatic phenotypes of *ls-tim* is its ability to enter diapause under cold conditions even in long summer photoperiods [10]. As this may be more adaptive in temperate regions where temperatures fall sharply in the autumn even when photoperiods are still relatively long, this might explain why *ls-tim* frequencies have spread from southern Italy both northwards and southwards generating a distance cline from the putative point of origin [10].

At the time this project was initiated, on Flybase, *cpo* was characterised by 6 transcripts. In most transcripts the first coding, exon 5, encoded 449 amino acids, but the isoform *cpo*-RH was the only one containing *cpo*^*Iso462Lys*^ which was located just downstream of the major splice junction in an extra stretch of exon 5 (S1 Fig). However, the modENCODE *Drosophila* developmental transcriptome project found no evidence for the existence of *cpo-RH* under the laboratory conditions in which the RNA was harvested [23]. Consequently, Cogni et al [11] have re-annotated *cpo*^*Iso462Lys*^, as *cpo*^*48034(A/T)*^ because current evidence suggests the SNP lies within an intron. Furthermore, Cogni et al (2014) have also re-annotated *cpo*^*Ala356Val*^ to *cpo*^*Ala347Val*^. We will from this point onwards refer to the two variants by their more recent re-annotations.

Nevertheless, it may be that the elusive *cpo-RH* isoform does indeed exist under the colder conditions that are more relevant for diapause induction, so we initially investigated this possibility. However our main focus has been to characterise whether these two *cpo* SNPs in European *D*. *melanogaster* populations reveal a latitudinal cline. *In(3R)Payne* is found at lower frequencies in temperate latitudes [19,20] so we might expect that in Europe, any cline observed in *cpo* variation would not be complicated by this linkage disequilibrium. In addition, and for the first time, we examine the effects of these *cpo* SNPs on diapause incidence while simultaneously controlling for their *timeless* background. Our results from Europe reveal a quite different scenario from that in North America.

## Results

### *cpo* variation in European populations

**Fig 1:**
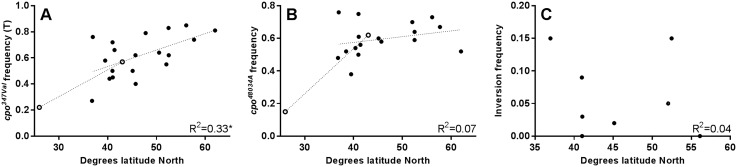
European frequencies of *cpo* polymorphisms and *In(3R)Payne*. Frequency of *cpo*^*347Val*^ in European populations plotted against latitude. Open circles, southern- and northern-most American populations [5], filled circles, European populations. * p<0.05. B) Frequency of *cpo*^*48034A*^ in European populations against latitude. C) Frequency of In(3R)Payne against latitude.

19 European populations were genotyped for *cpo*^*Ala347Val*^. (S1 Table). After applying Bonferroni correction for multiple testing in order to minimize the number of ‘false positives’, 5 populations out of 19 were not in Hardy-Weinberg equilibrium, generally due to the frequency of heterozygotes being lower than expected. Fig 1A shows how the allele frequencies changed over latitude. The data from Schmidt (2008) regarding the southern- and northernmost American populations are indicated by unfilled dots. In Europe, the allele *cpo*^*347Val*^ shows a trend similar to the one observed in the USA, increasing significantly in frequency with latitude (R^2^ = 0.33; p = 0.01). Both the homozygotes (*cpo*^*347Ala(C/C*)^ and *cpo*^*347Val(T/T)*^) show significant latitudinal clines (R^2^ = 0.22; p = 0.04 and R^2^ = 0.32; p = 0.01 respectively, not corrected for multiple testing), unlike the heterozygotes whose frequency does not change significantly with latitude (S2B Fig). The European and American trend lines show a similar slope, nevertheless the European data for *cpo*^*Ala347Val*^ are far more scattered than the corresponding data from North America (see Fig 4 in [5] where *cpo*^*Ala347Val*^ R^2^ = 0.92, p<0.00001). One possibility is that if *cpo*^*48034(A/T)*^ is the focal clinal polymorphism as suggested by Schmidt (2008), *cpo*^*Ala347Val*^ may not be as reliable a marker in Europe as in the USA for the putative *cpo*^*48034(A/T)*^ cline, if the linkage disequilibrium between the two sites is not as strong in Europe.

17 of the 19 European populations were also genotyped for *cpo*^*48034(A/T)*^ (S2 Table). Three of these populations were not in Hardy-Weinberg equilibrium, again because of a lack of heterozygous flies. Fig 1B shows the frequencies of the allele *cpo*^*48034A*^ which, contrary to expectations, does not vary significantly with latitude, giving a frequency around 0.6 in all localities. Similarly, the genotype frequencies do not show any significant latitudinal cline (S3 Fig).

Schmidt and co-workers (2008) determined the strong LD between the two SNPs (D = 0.216, p<0.0001) which is expected given that they are only 317 bp apart. LD was also studied in our European populations (S3 Table). Not surprisingly most populations show a very strong LD between the two sites, leading to an overall D’ value of 0.536 (0.480 when double heterozygotes are excluded from the analysis). LD is much stronger in the American populations, where D’ reaches a value 0.909 [5] and is likely the result of the bottleneck experienced by *D*. *melanogaster* upon their colonisation of the new continent, at most, a few hundred years ago [21].

The frequency of *In(3R)Payne* was also analysed in 9 European populations (S4 Table). As expected, given the temperate northern latitudes of Europe the frequency of inverted arrangements is very low in our dataset, (ranging from 0% to 15%, Fig 1C) and is in line with data collected at similar latitudes in USA, Asia and Australia [24]. The frequency does not vary significantly with latitude (R^2^ = 0.03), therefore the weak cline we have observed in Europe for *cpo*^*Ala347Val*^ is unlikely to be driven by the frequencies of this chromosomal inversion.

#### SNPs in *D*. *simulans*

**Fig 2:**
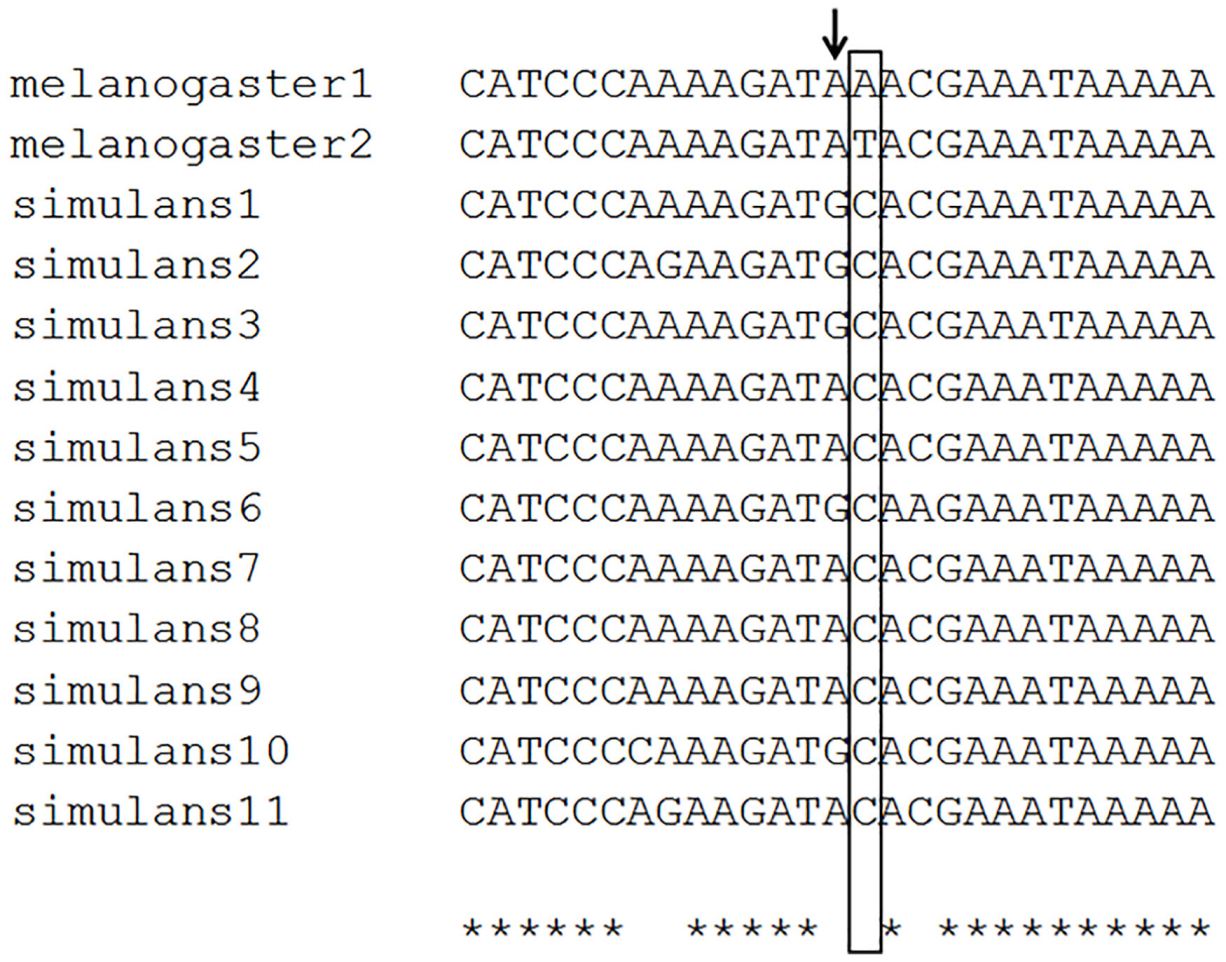
Nucleotide alignment of the *cpo* region in *D*. *melanogaster* and *D*. *simulans*. *D*. *melanogaster* sequences, top two rows. The box indicates the position of SNP *cpo*^*48034(A/T)*^ which appears to be monomorphic in *D*. *simulans*. The arrow points to a position which is polymorphic in *D*. *simulans* but not in *D*. *melanogaster*.

Schmidt and coworkers suggested that *cpo*^*347Val*^ and *cpo*^*48034A*^ represented the derived alleles [5] so we amplified and sequenced this *cpo* region in *D*. *simulans* flies captured in different locations from Africa. The sequences obtained suggest that site 347 is monomorphic in *D*. *simulans* and characterized by a *C* nucleotide (*cpo*^*Ala*^) confirming that *cpo*^*347Val*^ is the derived allele (Fig 2). Interestingly, the nucleotide at *cpo*^*48034(A/T)*^ is also represented by the *C* nucleotide only, but the nucleotide immediately upstream was found to be polymorphic *(A/G)* in *D*. *simulans*.

### Neutrality tests

In order to provide an initial indication of whether the *cpo* locus is under selection we applied the Tajima’s, Fu and Li’s and HKA test statistics to the region including the polymorphisms, and compared them to an intergenic region upstream of the polymorphic site. Table 1A shows the details of the DNA regions and flies that were analysed. An inspection of the alignment of the two genomic regions under study highlighted a remarkable number of insertion/deletion (indel) polymorphisms in the *cpo* region characterised by the two polymorphisms (Table 1B). Tajima’s D and Fu and Li’s test statistic [25] gave similar results (Table 1C) and generated significantly negative values, indicative of directional selection, only in the *cpo* region and only when indels are included in the analysis compared to the 5’ region. The Hudson-Kreitman-Aguadè test [26] was computed not including indel polymorphisms. Sequences from *D*. *simulans* retrieved from FlyBase were used for the interspecific comparison. As shown in Table 1D the results of the HKA test are not significant.

**Table 1:** *cpo* and *5’* sequences used for neutrality tests. A: details regarding the two genomic regions amplified, sequenced and analysed. Their length is reported in base pairs. N: number of individuals sequenced. Details of the four populations analysed. Latitude in degrees North. N: number of sequences analysed for the *5*’ and polymorphic *cpo* region (referred to as *5’* and *cpo* respectively). B: indel polymorphisms in the *cpo* and 5’ regions. Values calculated with DNAsp v5.10.01, using multiallelic model (all alleles are considered, also the ones which overlap in sequence). Mean indel length event is calculated averaging the length of every indel event in the alignment. Mean indel is calculated considering every indel event in each sequence. Values are in nucleotides. C. Results of the Tajima’s and Fu and Li’s test statistic. Results including and not including indels presented.* p<0.05, ** p<0.01. D. Results of the HKA test. Total number of sites in base pairs, gaps excluded S OBS: observed number of segregating sites (intraspecies comparison). S EXP: expected number of segregating sites (intraspecies comparison). DIFF OBS: observed number of differences (interspecies comparison). DIFF EXP: expected number of differences (interspecies comparison).

**A**						
DNA REGIONS						
Name	Location relative to SNP *cpo*^*Ala347Val*^				Length	N
5’	97 kb UPSTREAM				876	39
*cpo*	-				1401	34
LOCATIONS OF COLLECTION						
Code	Location			Latitude	N (5’)	N *(cpo)*
SP22	Nijar		Spain	36.97	11	10
BIT	Bitetto		Italy	41.02	6	5
TRV	Treviso		Italy	45.71	10	9
KOR	Korpilahti		Finland	62.02	12	10
**B**						
	TOTAL NUMBER INDELS EVENTS		MEAN INDEL LENGTH EVENT	MEAN INDEL LENGTH	NUMBER OF INDEL HAPLOTYPES	
5’	4		2.250	2.333	7	
*cpo*	21		14.524	6.922	21	
**C**						
	INDELS INCLUDED	TOT SITES	SEGREGATING SITES	TAJIMA’S D	D*	F*
5’	YES	876	38	0.579	0.618	0.670
	NO	868	30	0.077	0.301	0.191
*cpo*	YES	1401	292	-2.087*	-4.147**	-0.002**
	NO	1146	29	0.090	0.07	0.085
**D**						
	TOT SITES	S OBS	S EXP	DIFF OBS	DIFF EXP	χ^2^ (P-value)
5’	876	29	28.11	19.23	20.12	0.04 (0.84)
*cpo*	1142	27	27.89	21.88	20.99	

### Diapause in *cpo* variants

**Fig 3:**
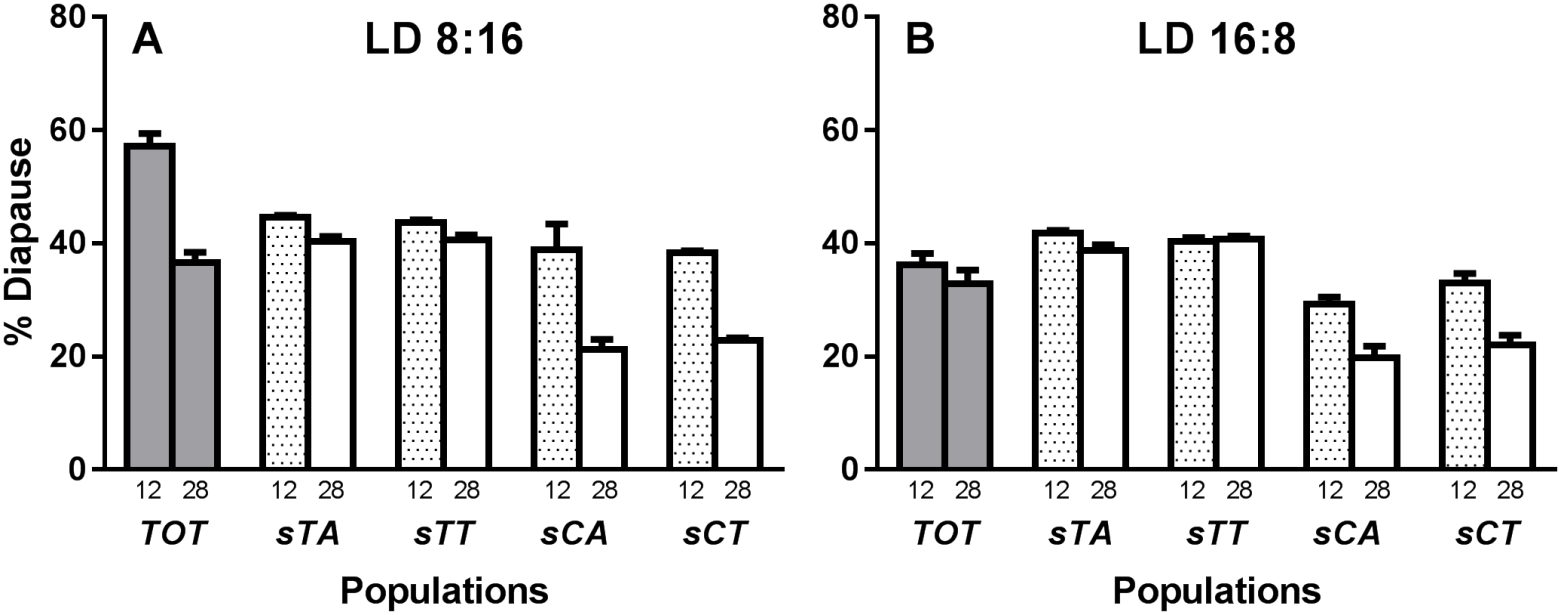
Diapause incidence in the Treviso population (TOT) and in the four sublines (*sTA*, *sTT*, *sCA* and *sCT*). Y axis: percentage diapause averaged among 5/6 replicates (±SEM). A) Diapause in LD 8:16 at 12 and 28 days (dotted and plain bars respectively). B) Diapause in LD 16:8. Gray bars: Treviso population. For a description of the genotypes of the populations see Table 2.

Table 2 shows the four different fly lines characterised by different allelic combinations of the two *cpo* SNPs that from a population from Treviso, Italy (Lat 45.71°N, Long 12.26°E) on a homozygous *s-tim* genetic background. The diapause results are shown in Fig 3 with the controls representing those flies maintained simultaneously in the dark (see Methods) as illustrated in S4 Fig. Diapause was also assessed in the polymorphic Treviso population (frequency of *ls-tim* = 0.5, referred to as ‘TOT’ in Fig 3, gray bars). Nevertheless, this population was not included in the statistical analysis. The result of the 4-way ANOVAs are shown in S5 Table. *cpo*^*Ala347Val*^ influences diapause levels (F_1,78_ = 321.1, p<<<0.01), whereas *cpo*^*48034(A/T)*^ has no significant effect (F_1,78_ = 1.40, p = 0.24). Furthermore, *cpo*^*Ala347Val*^ affects the way the diapause levels change with time (Days x SNP347 interaction F_1,78_ = 63.00, p<<<0.01). In particular the derived variant *cpo*^*347Val*^ increases diapause levels and maintains them at relatively high levels for a considerably longer than *cpo*^*347Ala*^. Surprisingly, *cpo*^*48034(A/T)*^ has no influence at all on any aspect of diapause in our studies.

**Table 2:** *tim* and *cpo* genotypes of the four lines created from a population from Treviso, Italy. The diapause incidence of these lines was then assessed at two different photoperiods (LD 8:16 and LD 16:8) and at two time points: after 12 and 28 days.

FLY LINE	*tim* ALLELE	*cpo*^*Ala347Val*^	*cpo*^*48034(A/T)*^
***sTA***	*s*/*s-tim*	*T/T cpo*^*347Val*^	*A/A*
***sTT***	*s*/*s-tim*	*T/T cpo*^*347Val*^	*T/T*
***sCA***	*s*/*s-tim*	*C/C cpo*^*347Ala*^	*A/A*
***sCT***	*s*/*s-tim*	*C/C cpo*^*347Ala*^	*T/T*

### cpo-RH isoform?

Given the lack of any phenotypic effect for the *cpo*^*48034(A/T)*^ site we sought to examine the expression of the *cpo-RH* transcript which carries this polymorphism under the colder conditions used to induce diapause. mRNA was harvested from the heads and the bodies of flies maintained at three different temperatures (12°C, 18°C and 25°C), at different time points (3 and 15 h after lights-on LD 12h:12h), and reverse transcribed to cDNA. Two PCR reactions were then performed to amplify either only isoform *cpo-RH* or all the isoforms except *cpo*-*RH*. *cpo*-*RH* could not be amplified in any of the aforementioned conditions.

## Discussion

*cpo* is a gene whose expression has been associated with diapause incidence not only in *D*. *melanogaster*, but in several other insects [27,28]. The direction of the change in its expression levels is not uniform among different species or developmental stages. *cpo* levels are low in diapausing *Sarcophaga crassipalpis* pupae [29], but *cpo* expression was found to be upregulated in diapausing *D*. *melanogaster* [5], *D*. *montana* [8,27] and in the mosquito *Culex pipiens* [28]. Recently, Schmidt and co-workers showed that different isoforms of *cpo* show opposite regulation in diapausing and non-diapausing ovaries of *D*. *melanogaster* [30].

Other than an RNA recognition motif (RRM), *cpo* also contains a conserved domain which shows similarity to type 1 antifreeze proteins (AFP) and/or Kv 1.4 voltage-gated potassium channels [31]. These proteins have been reported to be associated with cold tolerance in insects and spiders [32] and in cold hardening in an Antarctic midge [33]. Furthermore *cpo* contains ecdysone response elements, suggesting that its influence on diapause might be mediated by ecdysteroids [34].

Schmidt and colleagues (2008) observed that the derived alleles (*cpo*^*347Val*^ and *cpo*^*48034A*^ - although our *D*. *simulans* data could not confirm that *cpo*^*48034A*^ is derived as it has a *C* nucleotide at that position) both increased in frequency with latitude in the east coast of America, and suggested that *cpo*^*48034(A/T)*^ was the key clinal polymorphism, with *cpo*^*Ala347Val*^ acting as a marker. This (or these) relatively new alleles could thus confer evolutionary advantages at temperate latitudes, consistent with the Afrotropical origin of *D*. *melanogaster* [21], with selection favoring new mutations advantageous in northern habitats [19,35,36]. Indeed our neutrality tests of a 1.4 Kb region within *cpo* provided some, although not particularly compelling evidence for directional selection in European populations, but only when indels were included.

One way to confirm that a cline in allele frequency may be due to adaptation to the new environment is to evaluate the presence of the cline at different times, and correlate environmental changes with potential changes in the steepness of the allele frequency cline [37]. Alternatively, the presence of the same cline in different continents also supports the possibility that the polymorphism under study has an adaptive role. Many natural polymorphisms in *D*. *melanogaster* show a similar trend in frequency in different continents, including the indels in the *Insulin-like-Receptor* gene (although this gene also lies within *In(3R)Payne* [36]), chromosomal inversions [24], the number of Thr-Gly repeats in the *period* gene [38,39], as well as the very well-studied polymorphisms in the *Alcohol dehydrogenase* gene [40]. *cpo* has been identified as a Fst outlier in both American [12] and Australian populations [13] of *D*. *melanogaster*, thus suggesting that natural selection might be targeting this gene.

Lee and coworkers analysed the frequency of *cpo*^*48034(A/T)*^ in Australian populations, and found that *cpo*^*48034A*^ increased in frequency with latitude [18]. Nevertheless the SNP is in strong linkage disequilibrium with the chromosomal inversion *In(3R)Payne*, which shows a strong latitudinal cline in frequency in Australian populations [41]. The frequency of the inverted chromosome changes from 68% at a latitude of ~9°S, falling to zero above 40°S. When only standard chromosomes were considered in the analysis, the association between *cpo*^*48034A*^ and latitude ceased to be significant [18]. *In(3R)Payne* has been reported to vary significantly with latitude not only in Australian populations, but also in America and Asia [24] where again the frequency of inverted chromosomes is the highest close to the Equator, reaching values of around 10% at latitudes beyond 35°S.

In European fly populations, we have observed that *cpo*^*Ala347Val*^ shows a significant yet quite dispersed cline in allele frequency that was independent on the *In(3R)Payne* chromosomal inversion. We first thought that the ‘noisy’ cline emerged because we were assessing a marker for the *cpo*^*48034(A/T)*^ polymorphism, which Schmidt et al [5] had indicated was the important site for clinal selection. A weaker linkage disequilibrium between the two SNPs in European compared to American populations was also observed, suggesting that the bottleneck faced by *D*. *melanogaster* populations upon their recent colonisation of North America could have strengthened the LD between the two sites. However, on further study it was clear that *cpo*^*48034(A/T)*^ does not show any significant geographical variation in Europe.

Interestingly, several of the populations analysed were not in Hardy-Weinberg equilibrium and they were characterised by fewer heterozygotes than expected. A lack of heterozygotes can be attributed to subpopulation structure (the Wahlund effect). Alternatively, lack of heterozygotes can arise when disruptive or diversifying selection is acting on the population. As a consequence of the different selective pressures on a non-homogenous environment, disruptive selection acts against the heterozygotes, favouring the more extreme values of a trait. Schmidt and co-workers (2008) showed that flies characterised by one high- and one low-diapausing allele show a phenotype intermediate between the two homozygotes [5]. If *cpo*^*347Val*^ and *cpo*^*347Ala*^ are being selected in the North and in the South of Europe respectively, this could lead to a general decrease of heterozygotes whose phenotype is intermediate and therefore slightly disadvantageous at the extreme latitudes.

The attempt to amplify the *cpo-RH* isoform, the only one expressing *cpo*^*48034(A/T)*^, was unsuccessful at any of the temperatures/time points analysed. *cpo*-*RH*, is much shorter than the other isoforms and lacks the RNA binding domain, so it might be expressed only in conditions which were not assessed in our experiment. Alternatively it might only be expressed in a small subset of cells, or perhaps earlier in development thus making its identification problematic. Our negative results nevertheless resonate with those from the modENCODE project and a more recent paper by Schmidt and co-workers who performed a transcriptional profiling of diapause and were able to amplify the 12 new *cpo* isoforms, but not the isoform that is supposed to carry *cpo*^*48034(A/T*^ [30]. Consequently, at this point in time, it would appear that this polymorphism is to be found in non-coding DNA, at least under the conditions that Schmidt et al, modENCODE and ourselves have isolated the corresponding mRNA.

These negative results on finding *cpo-RH* may also illuminate the high level of variability found around this region that has also been reported by Kankare and colleagues [31] who compared the 3’ region of *cpo* exon 5 in five *D*. *virilis* group species. This variability is reflected in both *D*. *melanogaster* and *D*. *simulans* (Fig 2). Consequently at least three independent changes must be invoked to account for the variability between the sibling species at these adjacent sites, suggesting relaxed selection. Furthermore there was a lack of any significant effect on diapause of *cpo*^*48034(A/T)*^ when the different alternative alleles at the two *cpo* sites were placed on a natural standard *s-tim* background, in contrast to *cpo*^*347Val*^, which seems to significantly enhance diapause compared to *cpo*^*347Ala*^ in both 12 and 28 day observations.

Our results confirm that *cpo* is involved in regulating the diapause phenotype in European *D*. *melanogaster* flies and that the *cpo*^*Val347Ala*^ polymorphism shows a weak latitudinal cline that is considerably less impressive than its counterpart in North America. This may be because seasonal selection is stronger in North America than Europe, but more likely that the *cpo* polymorphisms in the former are in strong linkage disequilibrium with *In(3R)Payne* which exaggerates the cline. *In(3R)Payne* frequencies are extremely low in the European latitudes where we have collected our populations. These frequencies were not measured directly, but inferred from the frequencies of a polymorphism shown to be in complete linkage disequilibrium with the inversion in Australian fly populations (20). Further work to confirm the tightness of the LD between *In(3R)Payne* and the marker in European populations might be helpful. The intronic *cpo*^*48034(A/T)*^ variation originally suggested by Schmidt to be the focal polymorphism, has no effect on diapause when the *timeless* background is controlled, nor does it appear to show a latitudinal cline in Europe in spite of its close linkage with *cpo*^*Ala347Val*^. We suspect that *cpo*^*48034(A/T)*^ plays little or no role in diapause irrespective of which continent is studied. While our phenotypic analysis was based on a heterogeneous genetic background generated from a natural population from northern Italy, targeted mutagenesis using CRISPR/Cas9 at these two *cpo* sites on different genetic backgrounds would be required as definitive evidence that the upstream *cpo* polymorphism is indeed the focal, diapause-relevant *cpo* variant.

Pegoraro et al (submitted), have observed that in European fly populations, there is no clear latitudinal cline in diapause induction as has been documented in North America. Any cline in this phenotype in Europe is extremely weak as would be expected given the distributions of the *ls/s-tim* polymorphism, in which the newly arisen *ls-tim* allele which has enhanced levels of diapause is spreading from its proposed point of origin in southern Europe [10]. The weak *cpo*^*Ala347Val*^ cline that we see correlates, at least superficially, with the similarly weak phenotypic cline, suggesting perhaps that the residual diapause cline in Europe may be due to *cpo*^*Ala347Val*^. In any case, it is clear that in *D*. *melanogaster*, the *ls/s-tim* and *cpo*^*Ala347Val*^ polymorphisms play significant roles in diapause induction and any studies of seasonal ovarian arrest must take these polymorphisms into careful consideration when working with natural or laboratory populations.

## Materials and Methods

### Fly lines

Flies from natural *D*. *melanogaster* populations were collected from southern Spain (September 2008), various locations in southern and northern Italy (September 2008 for Treviso, October 2004 for all the others), Holland and Finland (September 2004 and September 2008 respectively, see S1 and S2 Tables). Fertilized females were isolated in single 2 x10 cm plastic vials containing fly food (4.6% sugar, 4.6% brewer’s yeast, 1.25% agar, 0.2% methyl 4-hydroxybenzoate) to establish a large number of isofemale lines from each population. From the time of collection, flies were maintained at 18°C in light-dark (LD) 12:12 cycles. Males were kept in ethanol and used for genotyping.

### Genotyping

*cpo*^*Ala347Val*^ and *cpo*^*48034(A/T)*^ were genotyped according to Schmidt and coworkers (2008). The DNA region under study was amplified with the primers *cpo-F* 5’-*AACATCCGTTGCTGCTGTC*-3’ and *cpo-R* 5’-*CCCCAAGCTGTCACTTTTGT*-3’. The following thermal profile was used to carry out the amplification: 40 cycles with 92°C for 35 sec, 55°C for 45 sec, 72°C for 30 sec. The PCR product was then subjected to treatment with the restriction enzyme *BsiEI*. The amplicons contain one *BsiEI* cutting site in *cpo*^*347T*^ (*cpo*^*347Val*^), and two sites in *cpo*^*347C*^ (*cpo*^*347Ala*^). The result of the digestion was then inspected in a 1.5% agarose gel. The amplicons were then sequenced and analysed with software Geospiza FinchTV Version 1.

A SNP in complete linkage disequilibrium with *In(3R)P* was identified by Anderson and coworkers [20] at position 12253813 of the *D*. *melanogaster* genome sequence (BDGP database). At this position standard and inverted chromosomes are characterised by an *A* or *C* respectively. This SNP was used as a marker for the inverted chromosome, and was genotyped with a Bi-PASA approach (Bidirectional PCR Amplification of Specific Alleles [42].

To detect and quantify the expression of *cpo* and in particular that of isoform *cpo-RH*, virgin flies from the Treviso population were subjected to the desired temperature (12, 18 and 25 degrees) and kept in light boxes in a 12:12 LD regime. After 4 days they were collected, their RNA was extracted and cDNA was synthesised. Specific primers were designed in order to amplify either only isoform RH, or all the isoforms except RH. The following common forward primer was used: *cpo-F* 5’*-AACATCCGTTGCTGCTGTC*-3’. As a reverse primer *cpo-R* 5’-*CCCCAAGCTGTCACTTTTGT*-3’ was used to amplify *cpo-RH*. The primer *cpo-R2* 5’-*ACGAAAAGTGTGCGAACCTC*-3’ recognises a region in exon 6, thus allowing the amplification of all the isoforms but *cpo-RH*. Primers for *Gapdh-glyceraldehyde 3-phosphate dehydrogenase* gene were used as internal controls. Their sequences were obtained from Schmidt et al., (2008).

### Line creation

Fly lines with different combinations of the two *cpo* SNPs were created using as a starting point a population obtained by combining equal numbers of non-virgin female flies from 35 isofemale lines collected in Treviso (Lat 45.71°N, Long 12.26°E). This location was chosen because it is characterised by an allelic frequency of ~50% for both SNPs. Single flies were genotyped using DNA obtained from the wings and then several flies (~10, 3^rd^ chromosomes) with the desired genotype were crossed to obtain the final lines. Consequently the *cpo* polymorphisms are studied on several genetic backgrounds that originated from 35 isofemale lines from Treviso, so any phenotype is averaged across this genetic diversity.

### Ovarian diapause

Male and female flies were collected within a six hour post-eclosion window and placed under two photoperiods: LD 8:16 and 16:8. They were maintained in 2 x10 cm plastic vials in 12.5 x19 x 26 cm light boxes containing a white fluorescent tube (standard T5 F4W/33) with an inbuilt heat sink and an electric fan (220V, 0.09A) to control the temperature. Temperature was monitored inside the chambers and maintained at 12.5+/-0.5°C within an incubator. Approximately 30 females from each vial were dissected in PBS, 12 or 28 days later, and their ovaries characterised according to King [43]. We used a stringent criterion so that a female was considered to be in reproductive arrest if its most advanced oocyte was pre-vitellogenic (prior to stage 8). The proportion of females in diapause from each vial represented an individual replicate, and at least 6 replicates were analysed for each population/photoperiod. As a thermal control, ovarian diapause was also tested simultaneously in ‘constant darkness (DD)’ by placing flies in additional vials covered in metal foil and exposed to the same LD cycle as the experimental groups within the same light boxes in the incubators. The diapausing proportion within each vial (replicate) was transformed to arcsin for ANOVA.

### Software

Linkage disequilibrium between the two polymorphic sites was calculated using R and the package “Genetics” (http://www.r-project.org). The Tajima’s test and the Fu’s and Li’s test statistics [25,44] were performed with NeutralityTest v1.1, kindly provided by Haipeng Li [44]. The Hudson-Kreitman-Aguadè test [26] was performed with DNAsp 5.10.01 (which does not consider gaps) [45], as the two loci were not exactly the same length.

## Supporting Information

S1 Fig *cpo* isoforms as of September 2008. Schematic representation of the six *cpo* splicing variants. Dark blue boxes encode the protein, whereas light blue ones represent the 5’ and 3’ UTR regions. The red and blue arrows represent the position of (then) SNP ‘A356V’ and SNP ‘48034 (A/T)’ respectively. The figure is not to scale and was redrawn from the database Flybase as it appeared in September 2008.
(JPG)

S2 Fig *cpo*^*A347V*^ genotype frequencies against latitude. Panels A, B and C show how *cpo*^*Ala347Val*^ genotype frequencies (*C/C*, *T/C*, *T/T* respectively), change with latitude of collection. *: p<0.05.
(JPG)

S3 Fig *cpo*^*48034(A/T)*^ genotype frequencies against latitude. Panels A, B and C show how the SNP *cpo*^*48034(A/T)*^ genotype frequencies (*A/A*, *A/T* and *T/T* respectively), change with latitude of collection.
(JPG)

S4 Fig Diapause incidence in the Treviso population (TOT) and in the four sublines (sTA, sTT, sCA and sCT) under constant darkness (DD). Y axis: percentage of diapause in two replicates per population (1 and 2). A) Diapause in LD 8:16. The two time points are compared (12 and 28 days, dotted and plain bars respectively). B) Diapause in 16:8, comparison between the two time points. Gray bars: Treviso population.
(JPG)

S1 Table Details of the flies used in the *cpo*^*Ala347Val*^ study. N: number of alleles analysed. Lat: latitude in degrees North. Long: longitude in degrees (Negative values: West; Positive values: East). Alt: altitude in meters above sea level. The last column shows the results of the Hardy-Weinberg test. *: p<0.01; ***: p<0.001.
(DOCX)

S2 Table Details of the flies used in the SNP *cpo*^*48034(A/T)*^ study. N: number of alleles analysed. Lat: latitude in degrees North. Long: longitude in degrees (Negative values: West; Positive values: East). Alt: altitude in metres above sea level. The last column shows the results of the Hardy-Weinberg test. *: p<0.05.
(DOCX)

S3 Table LD values calculated for each individual population and for the whole dataset. The analysis was carried out including the heterozygotes for both SNPs (DH, Double Heterozygotes), and excluding them from the dataset. When DH are included, observed frequency of each haplotype was estimated by the software, based on Maximum Likelihood The last row shows the results obtained by Schmidt and colleagues (2008) in American populations, using 75 extracted chromosomes (P. Schmidt, personal communication). N: number of haplotypes analysed. *: χ^2^>3.84; p<0.05; **: χ^2^>6.63; p<0.01; ***: χ^2^>10.80; p<0.001.
(DOCX)

S4 Table Frequency of *In(3R)Payne* in a subset of the European populations. N: number of alleles analysed. f(st) frequency of standard arrangements. f(in) frequency of inverted chromosomes. CL: 95% confidence limits calculated with the Wilson/Brown method.
(DOCX)

S5 Table Results of 4-way ANOVAs on diapause. LD: experimental samples. DD: controls. Significant values are indicated in red.
(DOCX)
