# Supplementary figures and images for: An Intronic Polymorphism in couch potato Is Not Distributed Clinally in European Drosophila melanogaster Populations nor Does It Affect Diapause Inducibility
Source: PLoS One. 2016 Sep 6;11(9):e0162370. doi: 10.1371/journal.pone.0162370 (PMC5012703; doi:10.1371/journal.pone.0162370)

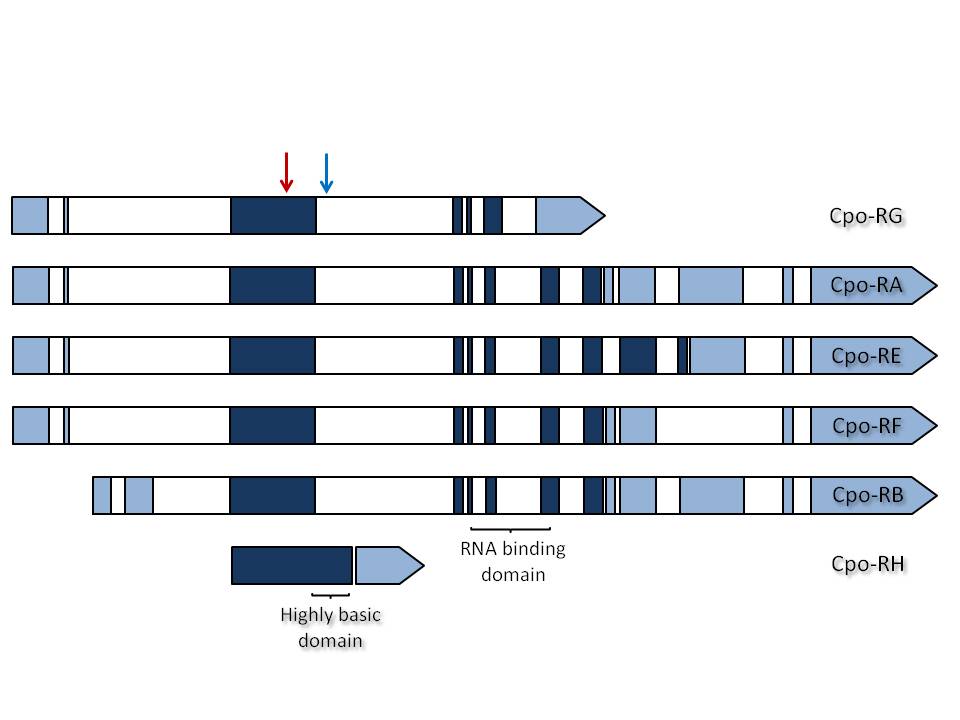

Supplement: S1 Fig — Schematic representation of the six cpo splicing variants. Dark blue boxes encode the protein, whereas light blue ones represent the 5’ and 3’ UTR regions. The red and blue arrows represent the position of (then) SNP ‘A356V’ and SNP ‘48034 (A/T)’ respectively. The figure is not to scale and was redrawn from the database Flybase as it appeared in September 2008. (JPG) [file pone.0162370.s001.jpg]

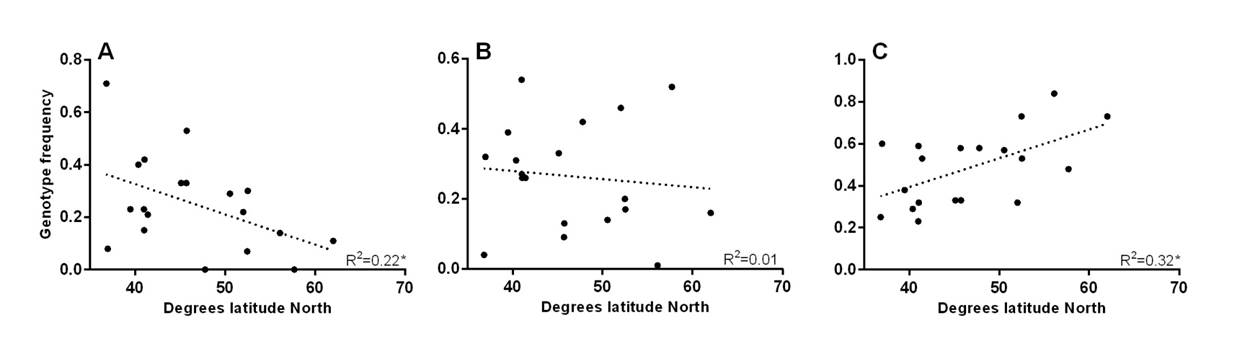

Supplement: S2 Fig — Panels A, B and C show how cpoAla347Val genotype frequencies (C/C, T/C, T/T respectively), change with latitude of collection. *: p<0.05. (JPG) [file pone.0162370.s002.jpg]

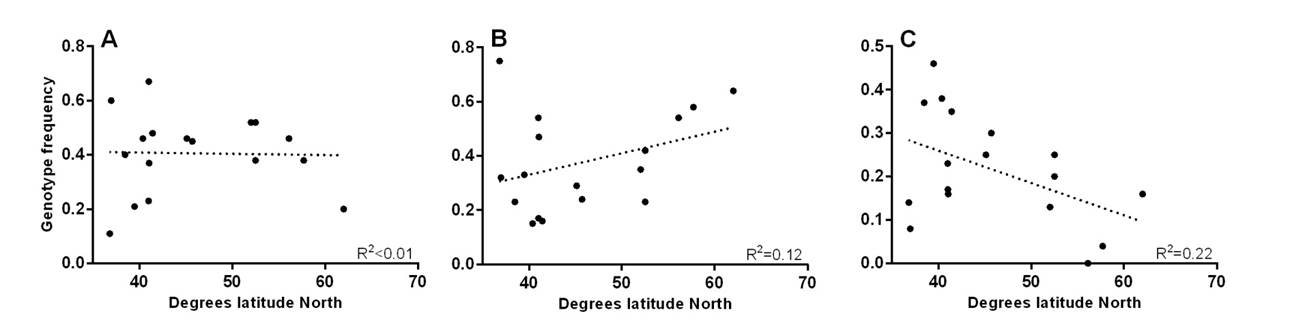

Supplement: S3 Fig — Panels A, B and C show how the SNP cpo48034(A/T) genotype frequencies (A/A, A/T and T/T respectively), change with latitude of collection. (JPG) [file pone.0162370.s003.jpg]

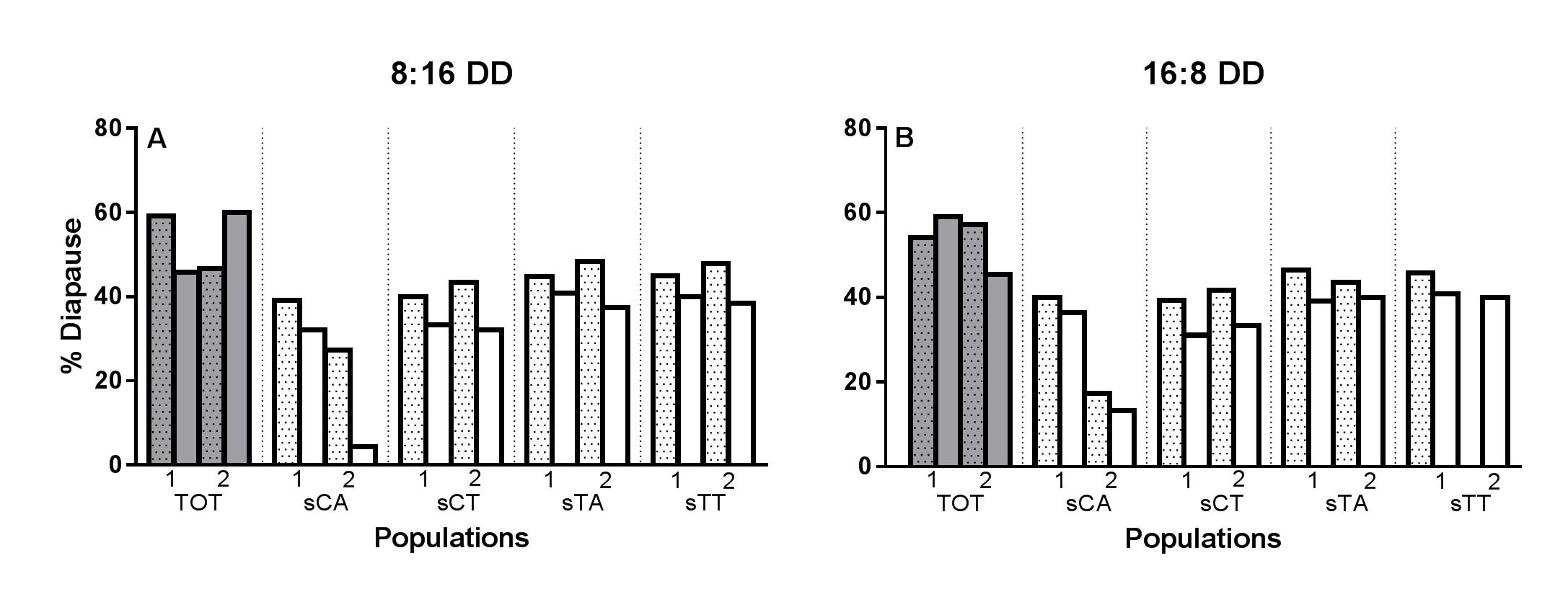

Supplement: S4 Fig — Y axis: percentage of diapause in two replicates per population (1 and 2). A) Diapause in LD 8:16. The two time points are compared (12 and 28 days, dotted and plain bars respectively). B) Diapause in 16:8, comparison between the two time points. Gray bars: Treviso population. (JPG) [file pone.0162370.s004.jpg]
