# Supplementary material for: An Intronic Polymorphism in couch potato Is Not Distributed Clinally in European Drosophila melanogaster Populations nor Does It Affect Diapause Inducibility
Source: PLoS One. 2016 Sep 6;11(9):e0162370. doi: 10.1371/journal.pone.0162370 (PMC5012703; doi:10.1371/journal.pone.0162370)
Supplement: S1 Table — N: number of alleles analysed. Lat: latitude in degrees North. Long: longitude in degrees (Negative values: West; Positive values: East). Alt: altitude in meters above sea level. The last column shows the results of the Hardy-Weinberg test. *: p<0.01; ***: p<0.001. (DOCX) [file pone.0162370.s005.docx]

| LINES DETAILS | | | | | | | FREQUENCIES | | | | | OBSERVED | | | EXPECTED | | |  |
| --- | --- | --- | --- | --- | --- | --- | --- | --- | --- | --- | --- | --- | --- | --- | --- | --- | --- | --- |
| LINE | LOCATION | COUNTRY | N | LAT | LONG | ALT | f(T) | f(C) | F(T/T) | f(T/C) | f(C/C) | # T/T | #T/C | #C/C | # T/T | #T/C | #C/C | χ^2^ |
| SP-14;15 | Dalias/Algarrobo | Spain | 56 | 36.82 | -2.87 | 423 | 0.27 | 0.73 | 0.25 | 0.04 | 0.71 | 7 | 1 | 20 | 2.01 | 10.98 | 15.01 | 23.13*** |
| SP-22 | Nijar | Spain | 50 | 36.97 | -2.21 | 345 | 0.76 | 0.24 | 0.60 | 0.32 | 0.08 | 15 | 8 | 2 | 14.44 | 9.12 | 1.44 | 0.38 |
| SP-38 | Requena | Spain | 194 | 39.49 | -1.10 | 716 | 0.58 | 0.42 | 0.38 | 0.39 | 0.23 | 37 | 38 | 22 | 32.33 | 47.34 | 17.33 | 3.78 |
| SAL | Salice | Italy | 70 | 40.38 | 17.38 | 48 | 0.44 | 0.56 | 0.29 | 0.31 | 0.40 | 10 | 11 | 14 | 6.86 | 17.27 | 10.86 | 4.61 |
| SP-44 | Vandeltormo | Spain | 70 | 40.99 | 0.08 | 478 | 0.50 | 0.50 | 0.23 | 0.54 | 0.23 | 8 | 19 | 8 | 8.75 | 17.50 | 8.75 | 0.26 |
| BIT | Bitetto | Italy | 82 | 41.02 | 16.75 | 149 | 0.72 | 0.28 | 0.59 | 0.27 | 0.15 | 24 | 11 | 6 | 21.23 | 16.55 | 3.23 | 4.61 |
| SP-43 | Alcaniz | Spain | 38 | 41.05 | -0.13 | 317 | 0.45 | 0.55 | 0.32 | 0.26 | 0.42 | 6 | 5 | 8 | 3.80 | 9.39 | 5.80 | 4.16 |
| SP-52 | S.Sadurni d’Anoia | Spain | 68 | 41.42 | 1.76 | 199 | 0.66 | 0.34 | 0.53 | 0.26 | 0.21 | 18 | 9 | 7 | 14.89 | 15.22 | 3.86 | 5.68 |
| CAV | Cavarzere | Italy | 78 | 45.13 | 12.08 | 3 | 0.50 | 0.50 | 0.33 | 0.33 | 0.33 | 13 | 13 | 13 | 9.75 | 19.50 | 9.75 | 4.33 |
| TRV | Treviso | Italy | 66 | 45.71 | 12.26 | 7 | 0.62 | 0.38 | 0.58 | 0.09 | 0.33 | 19 | 3 | 11 | 12.73 | 15.53 | 4.73 | 21.48*** |
| VIL | Villorba | Italy | 30 | 45.74 | 12.23 | 39 | 0.40 | 0.60 | 0.33 | 0.13 | 0.53 | 5 | 2 | 8 | 2.40 | 7.20 | 5.40 | 7.82 |
| VNN | Vienna | Austria | 24 | 47.79 | 16.36 | 183 | 0.79 | 0.21 | 0.58 | 0.42 | 0.00 | 7 | 5 | 0 | 7.52 | 3.96 | 0.52 | 0.83 |
| FUL | Fulda | Germany | 14 | 50.55 | 9.68 | 267 | 0.64 | 0.36 | 0.57 | 0.14 | 0.29 | 4 | 1 | 2 | 2.89 | 3.21 | 0.89 | 3.32 |
| HU | Houten | Holland | 74 | 52.03 | 5.17 | 3 | 0.55 | 0.45 | 0.32 | 0.46 | 0.22 | 12 | 17 | 8 | 11.36 | 18.28 | 7.36 | 0.18 |
| MAR | Market Harborough | England | 88 | 52.48 | -0.92 | 82 | 0.83 | 0.17 | 0.73 | 0.20 | 0.07 | 32 | 9 | 3 | 30.28 | 12.44 | 1.28 | 3.37 |
| KIL | Kilworth | England | 120 | 52.53 | 0.98 | 94 | 0.62 | 0.38 | 0.53 | 0.17 | 0.30 | 32 | 10 | 18 | 22.82 | 28.37 | 8.82 | 25.15*** |
| HΦJ | Hφjbjerg | Denmark | 138 | 56.11 | 10.21 | n.a. | 0.85 | 0.15 | 0.84 | 0.01 | 0.14 | 58 | 1 | 10 | 49.60 | 17.80 | 1.60 | 61.47*** |
| GOT | Goteborg | Sweden | 54 | 57.70 | 11.97 | 10 | 0.74 | 0.26 | 0.48 | 0.52 | 0.00 | 13 | 14 | 0 | 14.81 | 10.37 | 1.81 | 3.31 |
| KOR | Korpilahti | Finland | 110 | 62.02 | 25.55 | 104 | 0.81 | 0.19 | 0.73 | 0.16 | 0.11 | 40 | 9 | 6 | 36.00 | 16.99 | 2.00 | 12.17* |
