# Supplementary material for: An Intronic Polymorphism in couch potato Is Not Distributed Clinally in European Drosophila melanogaster Populations nor Does It Affect Diapause Inducibility
Source: PLoS One. 2016 Sep 6;11(9):e0162370. doi: 10.1371/journal.pone.0162370 (PMC5012703; doi:10.1371/journal.pone.0162370)
Supplement: S2 Table — N: number of alleles analysed. Lat: latitude in degrees North. Long: longitude in degrees (Negative values: West; Positive values: East). Alt: altitude in metres above sea level. The last column shows the results of the Hardy-Weinberg test. *: p<0.05. (DOCX) [file pone.0162370.s006.docx]

| LINES DETAILS | | | | | | | FREQUENCIES | | | | | OBSERVED | | | EXPECTED | | |  |
| --- | --- | --- | --- | --- | --- | --- | --- | --- | --- | --- | --- | --- | --- | --- | --- | --- | --- | --- |
| LINE | LOCATION | COUNTRY | N | LAT | LONG | ALT | f(A) | f(T) | F(A/A) | F(A/T) | f(T/T) | # A/A | #A/T | #T/T | #A/A | #A/T | #T/T | χ^2^ |
| SP-14;15 | Dalias/Algarrobo | Spain | 56 | 36.82 | -2.87 | 423 | 0.48 | 0.52 | 0.11 | 0.75 | 0.14 | 3 | 21 | 4 | 6.51 | 13.98 | 7.51 | 7.05 |
| SP-22 | Nijar | Spain | 50 | 36.97 | -2.21 | 345 | 0.76 | 0.24 | 0.60 | 0.32 | 0.08 | 15 | 8 | 2 | 14.44 | 9.12 | 1.44 | 0.38 |
| SP-28 | Jumilla | Spain | 60 | 38.48 | -1.32 | 520 | 0.52 | 0.48 | 0.40 | 0.23 | 0.37 | 12 | 7 | 11 | 8.01 | 14.98 | 7.01 | 8.52 |
| SP-38 | Requena | Spain | 48 | 39.49 | -1.10 | 716 | 0.38 | 0.63 | 0.21 | 0.33 | 0.46 | 5 | 8 | 11 | 3.38 | 11.25 | 9.38 | 2.00 |
| SAL | Salice | Italy | 26 | 40.38 | 17.38 | 48 | 0.54 | 0.46 | 0.46 | 0.15 | 0.38 | 6 | 2 | 5 | 3.77 | 6.46 | 2.77 | 6.20 |
| SP-44 | Vandeltormo | Spain | 70 | 40.99 | 0.08 | 478 | 0.50 | 0.50 | 0.23 | 0.54 | 0.23 | 8 | 19 | 8 | 8.75 | 17.50 | 8.75 | 0.26 |
| BIT | Bitetto | Italy | 24 | 41.02 | 16.75 | 149 | 0.75 | 0.25 | 0.67 | 0.17 | 0.17 | 8 | 2 | 2 | 6.75 | 4.50 | 0.75 | 3.70 |
| SP-43 | Alcaniz | Spain | 38 | 41.05 | -0.13 | 317 | 0.61 | 0.39 | 0.37 | 0.47 | 0.16 | 7 | 9 | 3 | 6.96 | 9.08 | 2.96 | 0.00 |
| SP-52 | S.Sadurni d’Anoia | Spain | 62 | 41.42 | 1.76 | 199 | 0.56 | 0.44 | 0.48 | 0.16 | 0.35 | 15 | 5 | 11 | 9.88 | 15.24 | 5.88 | 14.00* |
| CAV | Cavarzere | Italy | 48 | 45.13 | 12.08 | 3 | 0.60 | 0.40 | 0.46 | 0.29 | 0.25 | 11 | 7 | 6 | 8.76 | 11.48 | 3.76 | 3.65 |
| TRV | Treviso | Italy | 66 | 45.71 | 12.26 | 7 | 0.58 | 0.42 | 0.45 | 0.24 | 0.30 | 15 | 8 | 10 | 10.94 | 16.12 | 5.94 | 8.37 |
| HU | Houten | Holland | 46 | 52.03 | 5.17 | 3 | 0.70 | 0.30 | 0.52 | 0.35 | 0.13 | 12 | 8 | 3 | 11.13 | 9.74 | 2.13 | 0.73 |
| MAR | Market Harborough | England | 88 | 52.48 | -0.92 | 82 | 0.64 | 0.36 | 0.52 | 0.23 | 0.25 | 23 | 10 | 11 | 17.82 | 20.36 | 5.82 | 11.40* |
| KIL | Kilworth | England | 120 | 52.53 | 0.98 | 94 | 0.59 | 0.41 | 0.38 | 0.42 | 0.20 | 23 | 25 | 12 | 21.00 | 28.99 | 10.00 | 1.14 |
| HΦJ | Hφjbjerg | Denmark | 138 | 56.11 | 10.21 | n.a. | 0.73 | 0.27 | 0.46 | 0.54 | 0.00 | 32 | 37 | 0 | 36.96 | 27.08 | 4.96 | 9.26* |
| GOT | Goteborg | Sweeden | 52 | 57.70 | 11.97 | 10 | 0.67 | 0.33 | 0.38 | 0.58 | 0.04 | 10 | 15 | 1 | 11.78 | 11.44 | 2.78 | 2.51 |
| KOR | Korpilahti | Finland | 110 | 62.02 | 25.55 | 104 | 0.52 | 0.48 | 0.20 | 0.64 | 0.16 | 11 | 35 | 9 | 14.77 | 27.46 | 12.77 | 4.14 |
