# Supplementary material for: An Intronic Polymorphism in couch potato Is Not Distributed Clinally in European Drosophila melanogaster Populations nor Does It Affect Diapause Inducibility
Source: PLoS One. 2016 Sep 6;11(9):e0162370. doi: 10.1371/journal.pone.0162370 (PMC5012703; doi:10.1371/journal.pone.0162370)
Supplement: S3 Table — The analysis was carried out including the heterozygotes for both SNPs (DH, Double Heterozygotes), and excluding them from the dataset. When DH are included, observed frequency of each haplotype was estimated by the software, based on Maximum Likelihood The last row shows the results obtained by Schmidt and colleagues (2008) in American populations, using 75 extracted chromosomes (P. Schmidt, personal communication). N: number of haplotypes analysed. *: χ2>3.84; p<0.05; **: χ2>6.63; p<0.01; ***: χ2>10.80; p<0.001. (DOCX) [file pone.0162370.s007.docx]

| POPULATION | | | LD ESTIMATES | | | χ^2^ | |
| --- | --- | --- | --- | --- | --- | --- | --- |
| LOCATION | LAT | N | D | D’ | r |  | |
| Dalias/Algarrobo | **36.82** | 56 | 0.025 | 0.179 | 0.112 | 0.716 | **DH** |
|  |  | 54 | 0.023 | 0.173 | 0.106 | 0.612 |  |
| Nijar | **36.97** | 50 | 0.077 | 0.427 | 0.415 | 8.623** | **DH** |
|  |  | 44 | 0.051 | 0.317 | 0.304 | 4.082* |  |
| Jumilla | **38.48** | 60 | -0.148 | 0.680 | -0.596 | 21.264*** | **DH** |
|  |  | 54 | -0.140 | 0.653 | -0.564 | 17.147*** |  |
| Requena | **39.49** | 48 | 0.150 | 0.770 | 0.621 | 18.495*** | **DH** |
|  |  | 38 | 0.141 | 0.730 | 0.586 | 13.045*** |  |
| Salice | **40.38** | 26 | 0.134 | 0.581 | 0.538 | 7.532** | **DH** |
|  |  | 24 | 0.128 | 0.558 | 0.514 | 6.332* |  |
| Vandeltormo | **40.99** | 70 | -0.220 | 0.882 | -0.882 | 54.452*** | **DH** |
|  |  | 36 | -0.194 | 1 | -0.778 | 21.779*** |  |
| Bitetto | **41.02** | 12 | 0.091 | 0.497 | 0.473 | 5.365* | **DH** |
|  |  | 12 | 0.091 | 0.497 | 0.473 | 5.365* |  |
| Alcaniz | **41.05** | 38 | -0.148 | 0.836 | -0.607 | 14.007*** | **DH** |
|  |  | 32 | -0.133 | 0.809 | -0.553 | 9.784** |  |
| S. Sadurni d’Anoia | **41.42** | 110 | 0.012 | 0.125 | 0.063 | 0.434 | **DH** |
|  |  | 60 | 0.082 | 0.561 | 0.377 | 8.537** |  |
| Cavarzere | **45.13** | 48 | 0.080 | 0.634 | 0.402 | 7.745 | **DH** |
|  |  | 44 | 0.066 | 0.593 | 0.352 | 5.455 |  |
| Treviso | **45.71** | 66 | 0.141 | 0.650 | 0.585 | 22.613*** | **DH** |
|  |  | 62 | 0.135 | 0.631 | 0.564 | 19.758*** |  |
| Houten | **52.03** | 46 | 0.106 | 0.999 | 0.640 | 18.847*** | **DH** |
|  |  | 40 | 0.072 | 0.999 | 0.541 | 11.693*** |  |
| Market Harborough | **52.48** | 88 | 0.088 | 0.766 | 0.483 | 20.546*** | **DH** |
|  |  | 80 | 0.070 | 0.719 | 0.417 | 13.920*** |  |
| Kilworth | **52.53** | 120 | 0.120 | 0.550 | 0.503 | 30.349*** | **DH** |
|  |  | 104 | 0.104 | 0.497 | 0.448 | 20.830*** |  |
| Hφjbjerg | **56.11** | 138 | 0.030 | 0.273 | 0.187 | 4.800* | **DH** |
|  |  | 136 | 0.027 | 0.258 | 0.174 | 4.112* |  |
| Goteborg | **57.70** | 52 | 0.134 | 0.632 | 0.614 | 16.627*** | **DH** |
|  |  | 38 | 0.084 | 0.454 | 0.439 | 7.318** |  |
| Korpilahti | **62.02** | 110 | 0.012 | 0.125 | 0.063 | 0.434 | **DH** |
|  |  | 98 | 0.008 | 0.103 | 0.046 | 0.205 |  |
| ALL EUROPEAN POPULATIONS |  | 1138 | 0.105 | 0.536 | 0.457 | 237.840*** | **DH** |
|  |  | 950 | 0.090 | 0.480 | 0.398 | 149.540*** |  |
| ALL AMERICAN POPULATIONS |  | 75 | 0.216 | 0.909 | 0.909 | 37.986*** |  |
