# Supplementary material for: An Intronic Polymorphism in couch potato Is Not Distributed Clinally in European Drosophila melanogaster Populations nor Does It Affect Diapause Inducibility
Source: PLoS One. 2016 Sep 6;11(9):e0162370. doi: 10.1371/journal.pone.0162370 (PMC5012703; doi:10.1371/journal.pone.0162370)
Supplement: S4 Table — N: number of alleles analysed. f(st) frequency of standard arrangements. f(in) frequency of inverted chromosomes. CL: 95% confidence limits calculated with the Wilson/Brown method. (DOCX) [file pone.0162370.s008.docx]

| LINES DETAILS | | | | | | | **FREQUENCIES** | | **95% CL** | |
| --- | --- | --- | --- | --- | --- | --- | --- | --- | --- | --- |
| LINE CODE | LOCATION | COUNTRY | N | LAT | LONG | ALT | f(st) | f(in) | Upper | Lower |
| SP-22 | Nijar | Spain | 40 | **36.97** | -2.21 | 345 | 0.85 | 0.15 | 0.29 | 0.07 |
| SP-44 | Vandeltormo | Spain | 58 | **40.99** | 0.08 | 478 | 0.91 | 0.09 | 0.19 | 0.04 |
| BIT | Bitetto | Italy | 18 | **41.02** | 16.75 | 149 | 1.00 | 0.00 | 0.18 | 0.00 |
| SP-43 | Alcaniz | Spain | 60 | **41.05** | -0.13 | 317 | 0.97 | 0.03 | 0.11 | 0.01 |
| CAV | Cavarzere | Italy | 48 | **45.13** | 12.08 | 3 | 0.98 | 0.02 | 0.11 | 0.00 |
| HU | Houten | Holland | 66 | **52.03** | 5.17 | 3 | 0.95 | 0.05 | 0.13 | 0.01 |
| MAR | Market Harborough | England | 62 | **52.48** | -0.92 | 82 | 0.85 | 0.15 | 0.22 | 0.06 |
| HΦJ | Hφjbjerg | Denmark | 68 | **56.11** | 10.21 | n.a. | 1.00 | 0.00 | 0.05 | 0.00 |
