# Supplementary material for: An Intronic Polymorphism in couch potato Is Not Distributed Clinally in European Drosophila melanogaster Populations nor Does It Affect Diapause Inducibility
Source: PLoS One. 2016 Sep 6;11(9):e0162370. doi: 10.1371/journal.pone.0162370 (PMC5012703; doi:10.1371/journal.pone.0162370)
Supplement: S5 Table — LD: experimental samples. DD: controls. Significant values are indicated in red. (DOCX) [file pone.0162370.s009.docx]

|  | LD | | | | | DD | | | | |
| --- | --- | --- | --- | --- | --- | --- | --- | --- | --- | --- |
|  | SS | Deg F | MS | F | p | SS | Deg F | MS | F | p |
| days | 0.18317 | 1 | 0.18317 | 124.65 | 0.000000 | 0.05893 | 1 | 0.05893 | 5.970 | 0.026520 |
| 347 | 0.47185 | 1 | 0.47185 | 321.11 | 0.000000 | 0.12672 | 1 | 0.12672 | 12.839 | 0.002486 |
| 48934 | 0.00206 | 1 | 0.00206 | 1.40 | 0.240204 | 0.03501 | 1 | 0.03501 | 3.547 | 0.077967 |
| Photop | 0.02647 | 1 | 0.02647 | 18.02 | 0.000060 | 0.00005 | 1 | 0.00005 | 0.005 | 0.944900 |
| Days X 347 | 0.09258 | 1 | 0.09258 | 63.00 | 0.000000 | 0.00407 | 1 | 0.00407 | 0.412 | 0.529999 |
| Days X 48934 | 0.00173 | 1 | 0.00173 | 1.18 | 0.281472 | 0.00080 | 1 | 0.00080 | 0.081 | 0.779218 |
| 347 X 48934 | 0.00226 | 1 | 0.00226 | 1.54 | 0.218173 | 0.03209 | 1 | 0.03209 | 3.251 | 0.090245 |
| Days X Photop | 0.01219 | 1 | 0.01219 | 8.30 | 0.005128 | 0.00546 | 1 | 0.00546 | 0.553 | 0.467956 |
| 347 X Photop | 0.00505 | 1 | 0.00505 | 3.44 | 0.067556 | 0.00022 | 1 | 0.00022 | 0.022 | 0.882818 |
| 48934 X Photop | 0.00166 | 1 | 0.00166 | 1.13 | 0.291172 | 0.00036 | 1 | 0.00036 | 0.037 | 0.850199 |
| Days X 347 X 48934 | 0.00020 | 1 | 0.00020 | 0.14 | 0.710172 | 0.00059 | 1 | 0.00059 | 0.059 | 0.810508 |
| Days X 347 X Photop | 0.00275 | 1 | 0.00275 | 1.87 | 0.174905 | 0.00195 | 1 | 0.00195 | 0.198 | 0.662542 |
| Days X 48934 X Photop | 0.00015 | 1 | 0.00015 | 0.10 | 0.753184 | 0.00289 | 1 | 0.00289 | 0.292 | 0.596130 |
| 347 X 48934 X Photop | 0.00081 | 1 | 0.00081 | 0.55 | 0.460460 | 0.00086 | 1 | 0.00086 | 0.087 | 0.771603 |
| Days X 347 X 48934 X Photop | 0.00184 | 1 | 0.00184 | 1.25 | 0.266805 | 0.00298 | 1 | 0.00298 | 0.302 | 0.589999 |
| Error | 0.11462 | 78 | 0.00147 |  | | 0.15792 | 16 | 0.00987 |  | |
